# Inhibition of oxidative stress and apoptosis by camel milk mitigates cyclosporine‐induced nephrotoxicity: Targeting Nrf2/HO‐1 and AKT/eNOS/NO pathways

**DOI:** 10.1002/fsn3.2277

**Published:** 2021-04-05

**Authors:** Hany H. Arab, Ahmed H. Eid, Amany M. Gad, Rania Yahia, Ayman M. Mahmoud, Ahmed M. Kabel

**Affiliations:** ^1^ Department of Pharmacology and Toxicology College of Pharmacy Taif University Taif Saudi Arabia; ^2^ Department of Pharmacology Egyptian Drug Authority (EDA), formerly NODCAR Giza Egypt; ^3^ Department of Pharmacology and Toxicology Faculty of Pharmacy Sinai University El Ismailia Egypt; ^4^ Zoology Department, Faculty of Science Beni‐Suef University Beni‐Suef Egypt; ^5^ Biotechnology Department Research Institute of Medicinal and Aromatic Plants Beni‐Suef University Beni‐Suef Egypt; ^6^ Department of Pharmacology Faculty of Medicine Tanta University Tanta Egypt

**Keywords:** AKT, apoptosis, camel milk, cyclosporine, Nrf2, oxidative stress

## Abstract

Cyclosporine (CsA) is a widely used immunosuppressive agent that incurs marked nephrotoxicity in the clinical setting. Thus, there is a need for finding safe/effective agents that can attenuate CsA‐induced kidney injury. Meanwhile, the underlying mechanisms for CsA‐associated nephrotoxicity are inadequately investigated, in particular, the AKT/eNOS/NO pathway. Here, the present work aimed to explore the potential of camel milk, a natural product with distinguished antioxidant/anti‐inflammatory actions, to ameliorate CsA‐induced nephrotoxicity in rats. The molecular mechanisms related to renal oxidative aberrations and apoptosis were studied, including Nrf2/HO‐1 and AKT/eNOS/NO pathways. The kidney tissues were inspected using histopathology, ELISA, Western blotting, and immunohistochemistry. The present findings demonstrated that camel milk (10 ml/kg) significantly lowered creatine, BUN, and NGAL nephrotoxicity markers and the aberrant histopathology, with similar efficacy to the reference quercetin. Moreover, camel milk suppressed the renal oxidative stress, as evidenced by significantly lowering NOX‐1 and lipid peroxides and significantly augmenting the renal antioxidant moieties (GSH, GPx, and SOD), thereby, driving the restoration of Nrf2/HO‐1 pathway. Meanwhile, camel milk counteracted the pro‐apoptotic reactions by significantly lowering Bax protein expression, caspase‐3 activity/cleavage, and PARP cleavage, alongside significantly increasing the expression of the proliferation signal PCNA. Regarding the anti‐apoptotic AKT/eNOS/NO pathway, camel milk activated its signaling by significantly increasing the protein expression of PI3Kp110, p‐AKT(Ser473)/total AKT, and p‐eNOS (Ser1177)/total eNOS besides significantly boosting the renoprotective NO levels. In conclusion, these findings reveal that camel milk may be a promising candidate for the alleviation of CsA‐induced nephrotoxicity.

## Graphical abstract

Camel milk lowers creatinine, BUN, and NGAL nephrotoxicity markers. It suppresses oxidative stress, NOX‐1 expression, and lipid peroxides and activates Nrf2/HO‐1 pathway, and boosts GSH, GPx, and SOD antioxidant moieties. It curtails renal apoptosis markers (Bax, PARP, and caspase‐3) and upregulates PCNA, and activates the renal PI3K/Akt/eNOS/NO pathway.

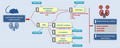

**Highlights**

- Camel milk lowers creatinine, BUN, and NGAL nephrotoxicity markers.
- It suppresses oxidative stress, NOX‐1 expression, and lipid peroxides.
- It activates Nrf2/HO‐1 pathway and boosts GSH, GPx, and SOD antioxidant moieties.
- It curtails renal apoptosis markers (Bax, PARP, and caspas‐3) and upregulates PCNA.
- It activates the renal PI3K/AKT/eNOS/NO pathway.

## 1 INTRODUCTION

*Cyclosporine* A (CsA) is a classical immunosuppressive agent that is commonly utilized for guarding against allograft rejection in patients with solid‐organ transplantation. In addition, CsA has been successfully used for the management of autoimmune disorders, such as rheumatoid arthritis and psoriasis (Ateyya, 2015; El‐Sheikh et al., 2019; Wu et al., 2018). However, the use of CsA has been associated with marked nephrotoxicity that culminates in chronic renal failure upon long‐term use. In this context, evolving evidence has revealed that organ‐transplantation patients undergoing long‐term CsA therapy have manifested chronic nephrotoxicity in the clinical setting (Wu et al., 2018). The hallmarks of the reported CsA‐evoked nephrotoxicity include tubular atrophy, inflammatory cell infiltration, arteriolopathy, tubular interstitial fibrosis, and exaggerated immunogenicity (Yoon & Yang, 2009).

The pathogenesis of CsA‐induced nephrotoxicity is multifactorial; however, oxidative stress plays a central role in the development and progression of this ailment. Virtually, the overshooting of the reactive oxygen species (ROS) and associated lipid peroxidation/oxidative aberrations are major hallmarks of CsA renal injury (Wu et al., 2018). In this regard, the overshooting of ROS has been characterized in CsA‐treated human renal mesangial cells (Vangaveti et al., 2021; Wu et al., 2018). Moreover, activated NADPH oxidase (NOX‐1) has been reported as a chief player in CsA‐evoked oxidative stress and aberrations. The most abundant ROS generated by CsA is the superoxide anion which is synthesized by the NOX‐1 enzyme. Moreover, an increase in plasma hydroperoxide levels has been reported in hypertensive patients undergoing CsA therapy (Calo et al., 2002; El‐Naga, 2014; Wu et al., 2018). Additionally, the decline of renal antioxidants, such as reduced glutathione has been described in LLC‐PK1 tubular cells (De Arriba et al., 2013; Wu et al., 2018).

Ample evidence has pointed to the role of apoptosis for mediating CsA‐induced renal injury. Studies have also pointed to the role of mitochondrial dysfunction in mediating CsA‐triggered renal tubular cell insult (De Arriba et al., 2013; Wu et al., 2018). The CsA‐evoked ROS production and diminished antioxidant defenses result in lowered mitochondrial membrane potential, increased mitochondrial pore permeability, and consequent release of the pro‐apoptotic cytochrome c to the intermembrane space, thereby, triggering the apoptotic machinery. In the context of apoptosis, phosphoinositide‐3‐kinase (PI3K)/protein kinase B (AKT) pathway has been characterized as a prosurvival pathway that orchestrates cellular growth and survival (Liu et al., 2020). Notably, interventions that can activate the PI3K/AKT signaling have been reported to effectively dampen the apoptotic events and renal injury in several renal pathologies (Arab et al., 2018a,b; Liu et al., 2020). The PI3K/AKT pathway has been reported to counteract endothelial cell apoptosis via upregulation of endothelial nitric oxide synthase (eNOS) that generates the vasodilator nitric oxide (Hasan et al., 2020). Of note, the role of the PI3K/AKT pathway in the pathogenesis of CsA‐evoked renal injury has been inadequately investigated.

Ample evidence has demonstrated that several antioxidant agents have exerted beneficial ameliorative effects against CsA‐evoked nephrotoxicity, with the advantage of incurring minimal adverse effects (Wu et al., 2018). Camel milk (CM) is unique milk in terms of its high content of antioxidants, including the potent antioxidant lactoferrin, alongside several antioxidant trace elements and vitamins (Abd‐Elhakim et al., 2020; Ayoub et al., 2018). Evolving evidence has confirmed the marked antioxidant features of CM in several experimental models, where it augments the glutathione peroxidase (GPx), reduced glutathione (GSH), superoxide dismutase (SOD), and total antioxidant capacity (Arab et al., 2018a,b). Virtually, CM is rich in vitamins C and A as well as potassium, calcium, magnesium, copper, zinc, and iron (Mohamed et al., 2019). CM possesses superior quality relative to the cow milk thanks to its lower lactose and cholesterol content, alongside the lack of lactose intolerance‐instigating agents, β‐casein and β‐lactoglobulin (Abd‐Elhakim et al., 2020). Additionally, CM contains multiple bioactive proteins, such as nanoantibodies and lactoperoxidase enzyme which mediate its remarkable antiviral/antibacterial and immune‐modulatory actions. Furthermore, CM has demonstrated insulin‐like activity which has been reported to save 30%–35% of the required dose of insulin for diabetic patients (Mohamed et al., 2019). Interestingly, CM has demonstrated favorable actions in the clinical and experimental setting against diabetes mellitus, wound healing (Ebaid et al., 2015), rheumatoid arthritis (Arab et al., 2017), steatohepatitis (Korish & Arafah, 2013), and diabetes mellitus‐ and toxicant‐induced renal pathologies (Arab et al., 2018a,b; Ayoub et al., 2018). Yet, its prospective ameliorative actions against CsA‐induced nephrotoxicity have not been studied. Hence, the present experimental work aimed to investigate the actions of CM against CsA‐evoked nephropathy and the underlying molecular mechanisms, pertaining to the oxidative and apoptotic events, including the Nrf2/HO‐1 and AKT/eNOS/NO pathways.

## 2 MATERIALS AND METHODS

### 2.1 Experimental animals

Male Wistar albino rats (*Rattus Norvegicus*; 8 weeks old; 180 ± 25 g) were provided by the Medical Research Center of King Fahd (Jeddah, KSA). All rats were maintained at controlled humidity (60 ± 10%), 25 ± 1°C temperature, and 12 hr/12 hr light/dark cycle. A one‐week acclimatization period was given to animals before starting the study. The rats had free access to the standard laboratory feeding pellets and ad libitum water for drinking.

### 2.2 Ethical statement

All the animal procedures were carried out according to the instructions stipulated by the Laboratory Animal Guide for Care and Use established by US‐NIH. The Research Ethical Committee of Taif University (Taif, KSA) has approved the current treatment protocol (Approval # 38‐35‐18).

### 2.3 Chemicals

Cyclosporine was procured from Sigma‐Aldrich Chemical Co. The camel milk was provided by Al‐Turath Al‐Saudia Co., Jeddah, KSA. It is a commercially available milk product with a standardized composition in terms of fat (3% w/v) and nonfat solids (6.3% w/v).

### 2.4 Experimental animal grouping and treatment protocol

A blinded technician randomly distributed the experimental animals into 7 groups (each group comprises 8 animals): Group I (control group): included rats that received s.c. olive oil (vehicle for cyclosporine) starting from the 1st day of the study till the 21st day; Group II (control + CM 10): included rats which received camel milk (2 ml/200g rat/day = 10 ml kg^−1^ day^−1^) by gavage for 21 days + s.c. olive oil for 21 days; Group III (CsA group): included rats that received s.c. injections of cyclosporine (CsA; 20 mg kg^−1^ day^−1^) for 21 days; Group IV (CsA + BM group): included rats which received s.c. cyclosporine (20 mg kg^−1^ day^−1^) + bovine milk (an isocaloric dose) by gavage for 21 days; Group V (CsA + CM 5 group): included rats which received s.c. cyclosporine (20 mg kg^−1^ day^−1^) + camel milk (5 ml kg^−1^ day^−1^) by gavage for 21 days; Group VI (CsA + CM 10 group): included rats which received s.c. cyclosporine (20 mg kg^−1^ day^−1^) + camel milk (10 ml kg^−1^ day^−1^) by gavage for 21 days; and Group VII (CsA + CM 5 group): included rats which received s.c. cyclosporine (20 mg kg^−1^ day^−1^) + the reference antioxidant quercetin (50 mg kg^−1^ day^−1^) by gavage for 21 days.

Animals were anesthetized with thiopental (50 mg/kg; i.p.) (Arab et al., 2021) and euthanized on the 22nd day, and the blood, as well as renal tissues, was immediately harvested. The selected experimental regimen agrees with previous studies (Abdel‐latif et al., 2013; Ateyya, 2015; El‐Sheikh et al., 2019; Mohamadin et al., 2005). The dose of the reference quercetin is based on previous studies (Liu et al., 2012), whereas the doses of camel milk are compliant with previous literature (Arab et al., 2017, 2018a,b).

### 2.5 Renal tissue preparation

At the end of the study period, one kidney (*n* = 4 per the experimental group) was fixed in 10% neutral‐buffered formalin for histopathology/immunohistochemistry studies. The other kidney was used for the assessment of the biochemical parameters and the Western blotting.

### 2.6 Assessment of the nephrotoxicity markers (creatinine, BUN, and NGAL)

Spectrophotometric diagnostic kits were used for the estimation of serum creatinine and blood urea nitrogen (BUN; Stanbio). Cusabio Biotech ELISA kit was used for the assay of renal neutrophil gelatinase‐associated lipocalin (NGAL; Cusabio Biotech, PRC), according to the instructions of the provider. The O.D. of the final color was measured at 450 nm. Each parameter was performed using 8 samples per group (one sample from each rat), and the assay was done in duplicate for each sample. The difference among groups was considered significant at *p* <.05.

### 2.7 Measurement of the oxidative damage/antioxidant defenses in renal tissues (NOX‐1, lipid peroxides, GSH, GPx, and SOD)

The renal content of NADPH oxidase‐1 (NOX‐1) was assayed using an ELISA kit provided by USCN Life Science, PRC, according to the provider's instructions. The assay of renal lipid peroxides expressed as malondialdehyde (MDA) was carried out according to the assay established by Buege and Aust (1978). The levels of the renal reduced glutathione (GSH) were assayed using DTNB (Ellman's reagent), as previously described by Beutler et al. (1963), whereas the measurement of renal glutathione peroxidase (GPx) was assayed as established by Paglia & Valentine (1967). Each parameter was performed using 8 samples per group and the assay was done in duplicate for each sample. The difference among groups was considered significant at *p* <.05.

### 2.8 Measurement of renal Nrf2 and HO‐1

The assay of the renal heme oxygenase 1 (HO‐1) and nuclear factor (erythroid‐derived 2)‐like 2 (Nrf2) was carried out with the aid of MyBioSource ELISA kits. For Nrf2 determination, the extraction of the nuclear proteins was performed using Cayman nuclear extraction kit (Cayman Chemical), followed by the assay of Nrf2. Strict adherence to the provider's instructions was followed. The final color of the above assays was measured at 450 nm. Each parameter was performed using 8 samples per group, and the assay was done in duplicate for each sample. The difference among groups was considered significant at *p* <.05.

### 2.9 Determination of the renal caspase‐3 activity

The assay of caspase‐3 activity was carried out with the aid of Sigma‐Aldrich corresponding assay kit and the final color was read at 405 nm. The determination of caspase‐3 was performed using 8 samples per group, and the assay was done in duplicate for each sample. The difference among groups was considered significant at *p* <.05.

### 2.10 Determination of the renal nitric oxide (NO) levels

The assay of total nitric oxide was performed as established by Miranda et al. (2001), where the nitrite/nitrate stable metabolites were measured following the protein precipitation of the samples. Vanadium trichloride (0.8% in 1 M HCl) triggered the reduction of nitrate to nitrite and Greiss reagent was added for final color development, which was measured at 540 nm (Optima SP‐3000, Japan). The determination of NO was performed using 8 samples per group, and the assay was done in duplicate for each sample. The difference among groups was considered significant at *p* <.05.

### 2.11 Western blotting

The protein lysates of the renal tissues were prepared by homogenization in RIPA buffer complemented with protease/phosphatase inhibitors cocktail (Arab et al., 2021). Equal amounts of renal protein lysates were loaded to SDS polyacrylamide gels for separation, and the resolved proteins were transferred to polyvinylidene difluoride (PVDF) membranes. 5% BSA/1X TBS was used for blocking the membranes, and the primary antibodies were applied overnight at 4°C: anti‐cleaved poly (ADP‐ribose) polymerase (PARP), anti‐cleaved caspase‐3 (Asp175), anti‐phospho‐eNOS (Ser1177), anti‐total eNOS, anti‐phospho‐AKT (Ser473), anti‐total AKT, anti‐PI3K p110α, and the loading control anti‐β actin (Cell Signaling Technology). Following the washing in TBS‐T and treatment of membranes with horseradish peroxidase‐tagged secondary antibodies (Cell Signaling Technology), the antigen–antibody protein complexes were detected with Biorad Clarity Western ECL substrate. The intensity of the final protein bands was quantified with the aid of Image J software (Bethesda). The data of the immunoblotting were extracted from 3 independent samples from each group. The difference among groups was considered significant at *p* <.05.

### 2.12 Hematoxylin and eosin staining

Following the fixation of kidney specimens in 10% neutral‐buffered formalin and embedding in paraffin, the specimens were cut with a microtome (Leica Microsystems) to 4 µm sections. A routine staining protocol was followed using hematoxylin and eosin (H&E) (Arab et al., 2020). A blinded pathologist to the identity of specimens examined the slides under light microscope (Leica Microsystems GmbH). The histology was done on 4 samples from each group.

### 2.13 Immunohistochemistry

Four‐micrometer sections were used for the immunohistochemical labeling of target proteins. Blocking of the sections was performed with 5% BSA for 2 hr, as reported (Arab et al., 2021; Fikry et al., 2019). Primary antibody incubation was carried out overnight at 4°C in a moist chamber using anti‐PCNA and anti‐Bax antibodies (Thermo Scientific). After washing of the tissue sections, the HRP‐labeled secondary antibodies were applied, followed by antigen–antibody protein complex detection using DAB (Sigma‐Aldrich) and section counterstaining with hematoxylin. A blinded slide examination protocol was followed to avoid bias. The immune‐intensity of the stained proteins was quantified with the aid of Image J software (Bethesda). The immunohistochemistry was done on 4 samples from each group. The difference among groups was considered significant at *p* <.05.

### 2.14 Statistical analysis

Before proceeding to the statistical comparisons, the normality of data was checked through the Shapiro–Wilk normality test. The statistical comparison among experimental groups was executed using one‐way analysis of variance and Tukey–Kramer multiple comparisons (SPSS Software; version 17). *p* <.05 was regarded as the minimally accepted significance level, and the data were expressed as mean ± *SEM*.

## 3 RESULTS

### 3.1 Camel milk improves serum creatinine, blood urea nitrogen (BUN), and renal neutrophil gelatinase‐associated lipocalin (NGAL) injury markers

**FIGURE 1:**
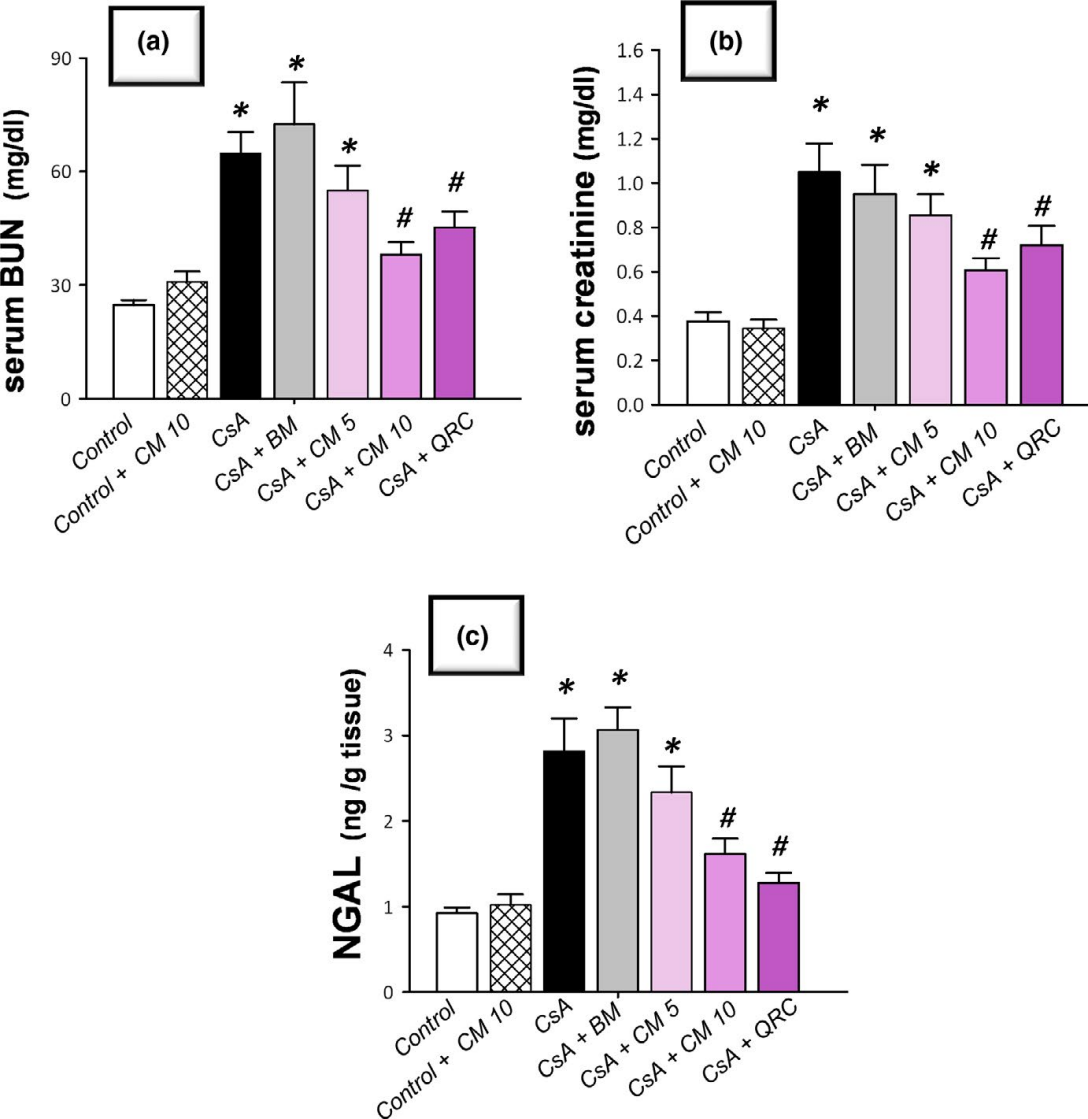
Camel milk ameliorates the renal injury markers in cyclosporine‐induced nephrotoxicity in rats. (a) The levels of serum creatinine. (b) The levels of blood urea nitrogen (BUN). (c) Renal protein expression of neutrophil gelatinase‐associated lipocalin (NGAL). The values are mean ± *SEM*, for *n* = 8 per each group. *Significant versus control gp at *p* <.05; *^#^*Significance versus CsA‐treated gp at *p* <.05. BM, an isocaloric dose of bovine milk; CsA, Cyclosporine; CM 5, 5 ml kg^−1^ day^−1^ dose of camel milk; CM 10, 10 ml kg^−1^ day^−1^ dose of camel milk; QRC, 50 mg kg^−1^ day^−1^ dose of the reference antioxidant quercetin

The CsA‐triggered nephrotoxicity was investigated by measuring serum BUN and creatinine along with the sensitive renal tubular injury marker NGAL. As depicted in Figure 1, CsA instigated marked nephrotoxicity, as evidenced by significant elevation of serum creatinine and BUN, and renal NGAL levels to reach 2.8‐fold, 2.6‐fold, and 3.1‐fold, respectively, relative to the control group. Regarding the ameliorative effects of camel milk, it elicited a dose‐dependent decline of these nephrotoxicity markers, relative to the CsA‐treated group. Of note, even though the lower dose of camel milk (5 ml kg^−1^ day^−1^) elicited a lowering of these biomarkers, the decline did not attain statistical significance. More importantly, the 10 mg kg^−1^ day^−1^ dose afforded an obvious and statistically significant lowering of serum creatinine, BUN, and NGAL levels by 42.2%, 41.4%, and 54.6%, respectively, relative to the CsA‐treated group. Hence, the dose of 10 mg kg^−1^ day^−1^ was chosen for the next set of experiments to dissect the impact of camel milk on oxidative stress and apoptotic events in the renal tissues. Interestingly, the ameliorative effects of the 10 ml/kg dose of camel milk were close to the elicited effects of the reference antioxidant quercetin, signifying the efficacy of camel milk for the attenuation of renal injury. Notably, the use of an isocaloric dose of bovine milk was not able to demonstrate significant improvement in serum nephrotoxicity markers, indicating that the distinctive camel milk composition is the main determinant for the observed improvement of renal function, rather than the caloric content.

### 3.2 Camel milk attenuates cyclosporine‐evoked renal histopathologic damage

**FIGURE 2:**
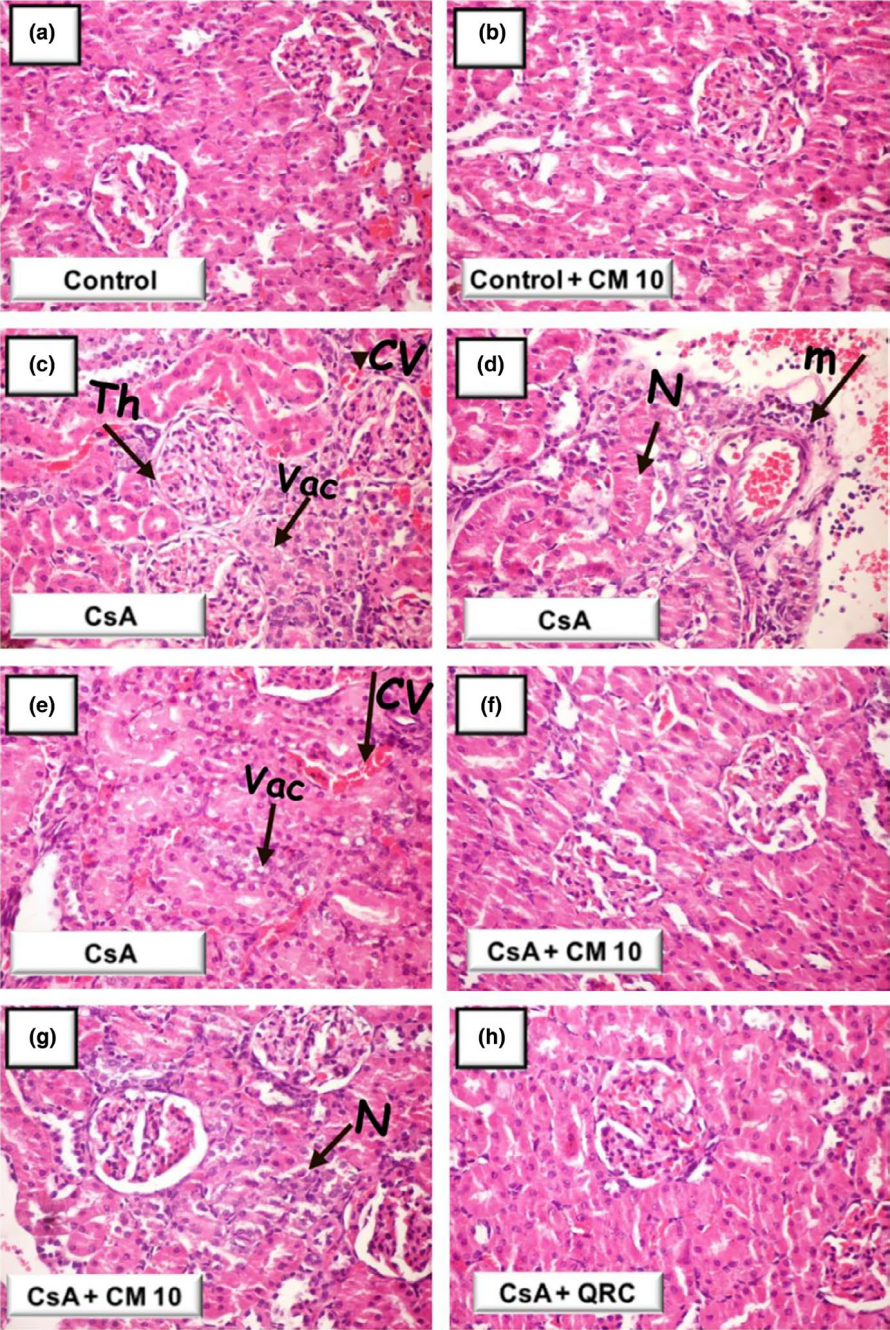
Camel milk ameliorates cyclosporine‐evoked renal histopathologic damage in rats. Kidney samples were stained using hematoxylin–eosin (×200 magnification) for exploring the renal aberrations. (a, b) The control and camel milk‐treated control groups revealed intact histologic features of the renal parenchyma. (c–e) The cyclosporine‐treated group showed marked renal damage, including slight thickening (Th) of Bowman's capsule, congestion of the glomerular tufts, and necrobiotic alterations of the renal tubular epithelium (N). Furthermore, an obvious influx of inflammatory cells (m), congestion of the intertubular blood capillaries (CV), and vacuolation (Vac) of tubular epithelium lining were detected. (f, g) Camel milk protected against these pathologic changes and preserved the renal architecture with the detection of focal resolving areas of renal tubular necrosis (N). (h) Quercetin attenuated the histopathologic changes. CsA, Cyclosporine; CM 10, 10 ml kg^−1^ day^−1^ dose of camel milk; QRC, 50 mg kg^−1^ day^−1^ dose of the reference antioxidant quercetin

To further characterize the favorable effects of camel milk on cyclosporine‐evoked renal damage, the microscopic damage/aberrations were examined by histopathology (Figure 2). At the microscopic level, the examination of the kidney tissues demonstrated that the control and camel milk‐treated control groups showed intact histologic features of renal parenchyma (Table 1 and Figure 2a,b). Conversely, the cyclosporine‐treated group manifested severe renal damage and histologic changes, such as slight thickening of Bowman's capsule, necrobiotic alterations of the renal tubular epithelium, and congestion of the glomerular tufts. Additionally, marked recruitment of inflammatory cells, congestion of the intertubular blood capillaries, and vacuolation of the tubular epithelium lining were observed (Table 1 and Figure 2c–e). Interestingly, camel milk protected against these pathologic alterations and preserved the renal architecture (Table 1 and Figure 2f,g). Additionally, camel milk‐treated rats displayed areas of focal resolving necrosis of renal tubules. Likewise, quercetin attenuated CsA‐evoked pathologic changes (Table 1 and Figure 2h). In line with the alleviation of renal dysfunction biomarkers, camel milk and quercetin mitigated the CsA‐induced renal histopathologic damage.

**TABLE 1:** Renal histopathologic damage in cyclosporine‐evoked nephrotoxicity in rats

Histopathologic alteration	Control	Control + CM 10	CsA	CsA + CM 10	CsA + QRC
Focal tubular necrosis	−	−	++	+	−
Vacuolation of tubular epithelium	−	−	+++	−	−
Congestion of intertubular blood capillaries	−	−	+++	−	−
Congestion and hypertrophy of glomerular tuft	−	−	++	−	−

Note

(+++) describes severe damage where >35% of the examined tissue sections is affected; (++) describes moderate damage where 15%–35% of the examined tissue sections is affected; (+) describes mild change where <15% of the examined tissue sections is affected; (−) indicates a lack of histologic change.

### 3.3 Camel milk diminishes renal oxidative stress and NOX‐1 expression, restores Nrf2/HO‐1 pathway, and augments the antioxidant defenses

**FIGURE 3:**
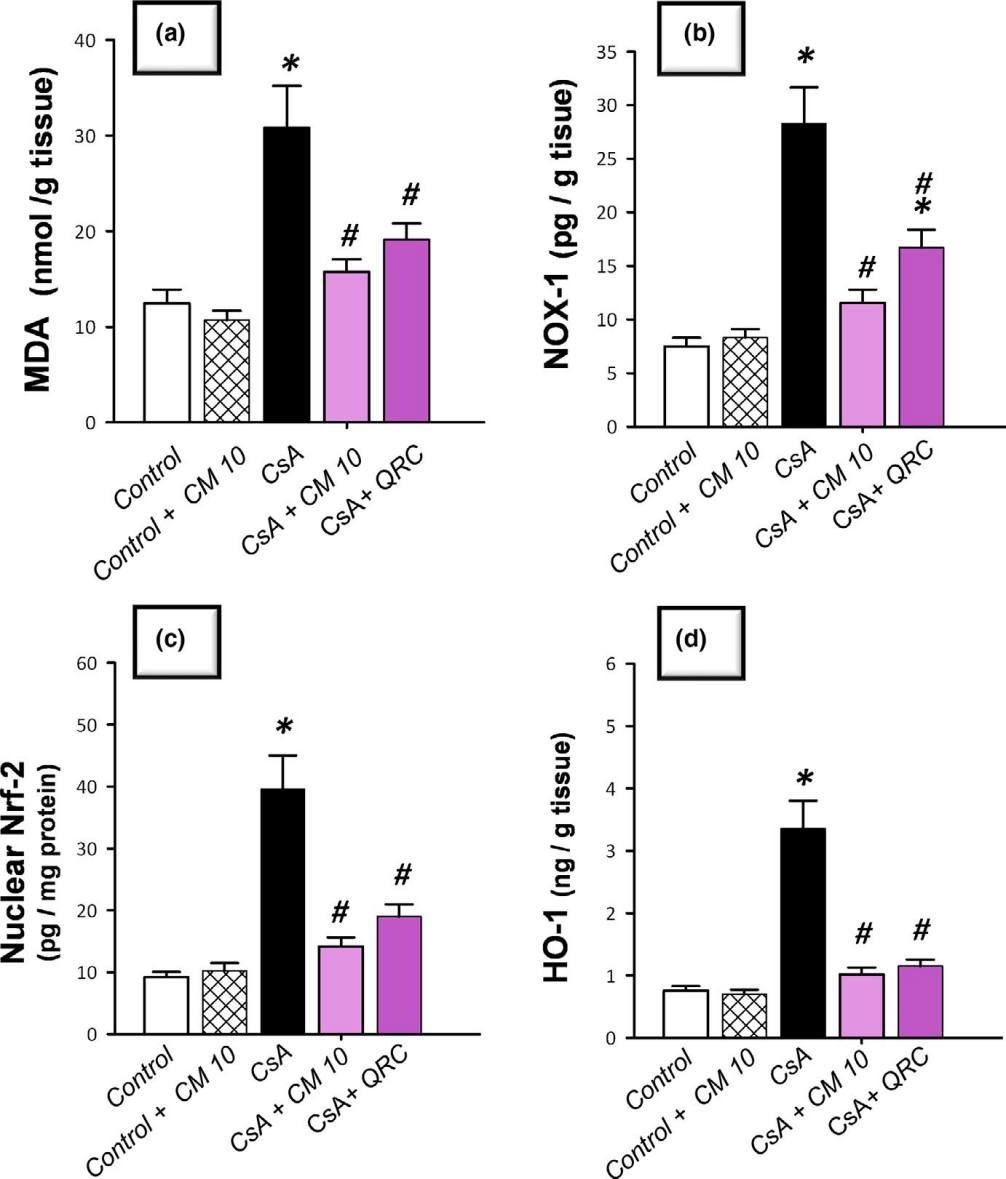
Camel milk combats the renal oxidative stress, increases NOX‐1 expression, and restores the Nrf2/HO‐1 pathway in cyclosporine‐induced nephrotoxicity in rats. (a) The levels of lipid peroxides. (b) The levels of NADPH oxidase‐1 (NOX‐1). (c) The levels of nuclear factor erythroid 2‐related factor‐2 (Nrf2) in the nuclear fraction. (d) Heme oxygenase‐1 (HO‐1) levels. The values are mean ± *SEM*, for *n* = 8 per each group. *Significant versus control gp at *p* <.05; *^#^*Significance versus CsA‐treated gp at *p* <.05. CsA, Cyclosporine; CM 10, 10 ml kg^−1^ day^−1^ dose of camel milk; QRC, 50 mg kg^−1^ day^−1^ dose of the reference antioxidant quercetin

**FIGURE 4:**
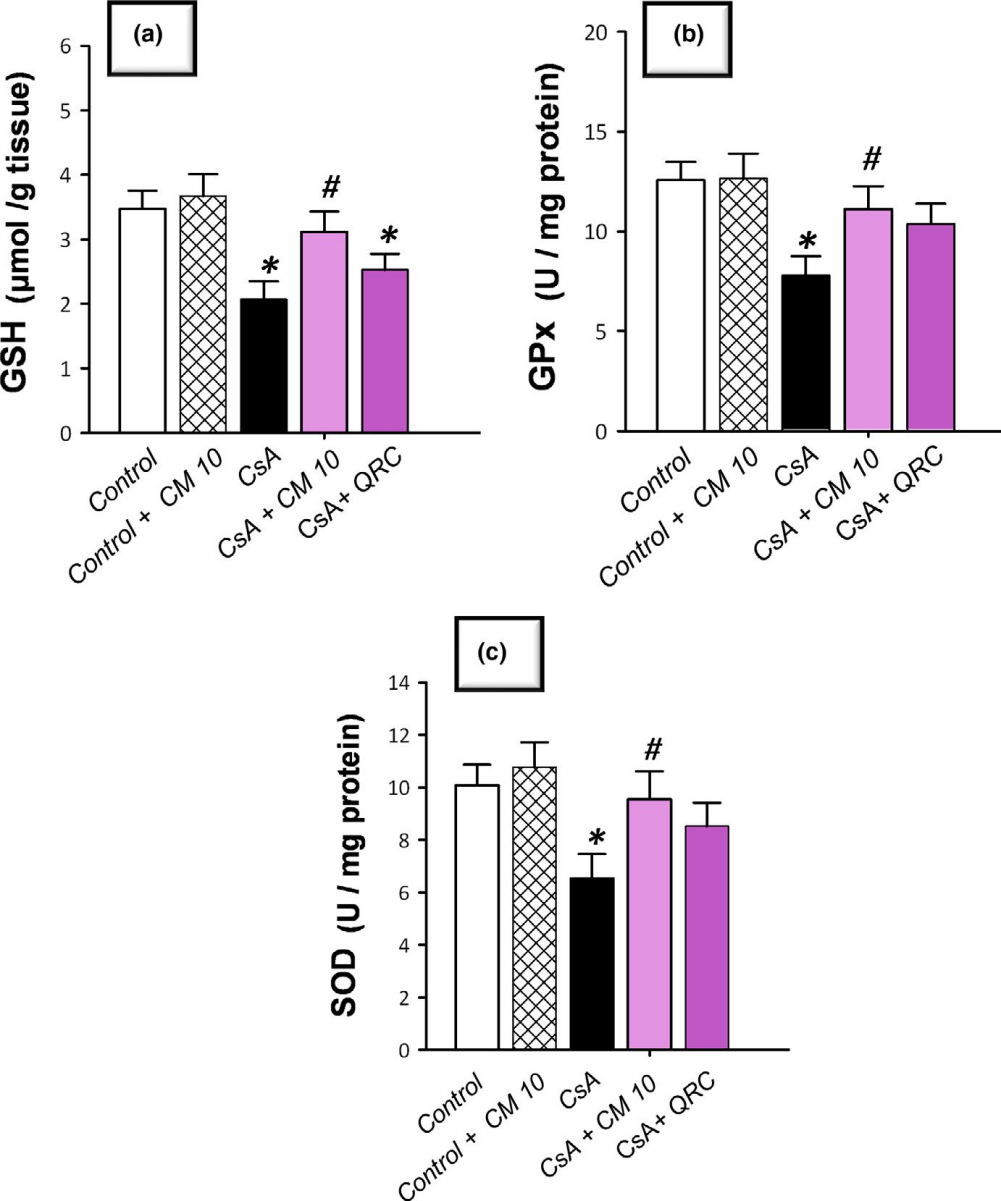
Camel milk augments the antioxidant armory in cyclosporine‐induced nephrotoxicity in rats. (a) The levels of reduced glutathione (GSH). (b) The levels of glutathione peroxidase (GPx). (c) The levels of superoxide dismutase (SOD). The values are mean ± *SEM*, for *n* = 8 per each group. *Significant versus control gp at *p* <.05; *^#^*Significance versus CsA‐treated gp at *p* <.05. CsA, Cyclosporine; CM 10, 10 ml kg^−1^ day^−1^ dose of camel milk; QRC, 50 mg kg^−1^ day^−1^ dose of the reference antioxidant quercetin

The redox status of renal tissues was explored via detecting the lipid peroxide levels and the pro‐oxidant NADPH oxidase‐1 (NOX‐1) expression together with the antioxidant defenses, such as reduced glutathione (GSH), glutathione peroxidase (GPx), and superoxide dismutase (SOD). Additionally, Nrf2/HO‐1 pathway was investigated by detecting Nrf2 and HO‐1 protein expression. As demonstrated in Figure 3, CsA significantly increased the levels of renal lipid peroxides (MDA) and NOX‐1 to reach 2.5‐fold and 3.8‐fold, respectively, relative to the control group. Notably, a compensatory activation of the Nrf2/HO‐1 pathway was observed in response to the CsA insult, as evidenced by a significant increase of the nuclear Nrf2 and cellular HO‐1 levels that reached 4.3‐fold and 4.4‐fold, respectively, relative to the control group. Regarding the antioxidant machinery, CsA significantly diminished the renal GSH, GPx, and SOD to reach 0.49‐fold, 0.62‐fold, and 0.59‐fold, respectively, relative to the control group (Figure 4). Camel milk diminished the renal oxidative stress, and thus, returned the Nrf2/HO‐1 pathway signaling to the normal values. This was evidenced by counteracting the changes of the oxidative stress markers and restoration of the protein expression of Nrf2 and HO‐1 signals back to their normal values (Figure 3). Equally important, camel milk opposed the CsA‐induced decline of the renal antioxidant defenses (Figure 4).

### 3.4 Camel milk curtails the renal pro‐apoptotic events and enhances the renal cellular survival

**FIGURE 5:**
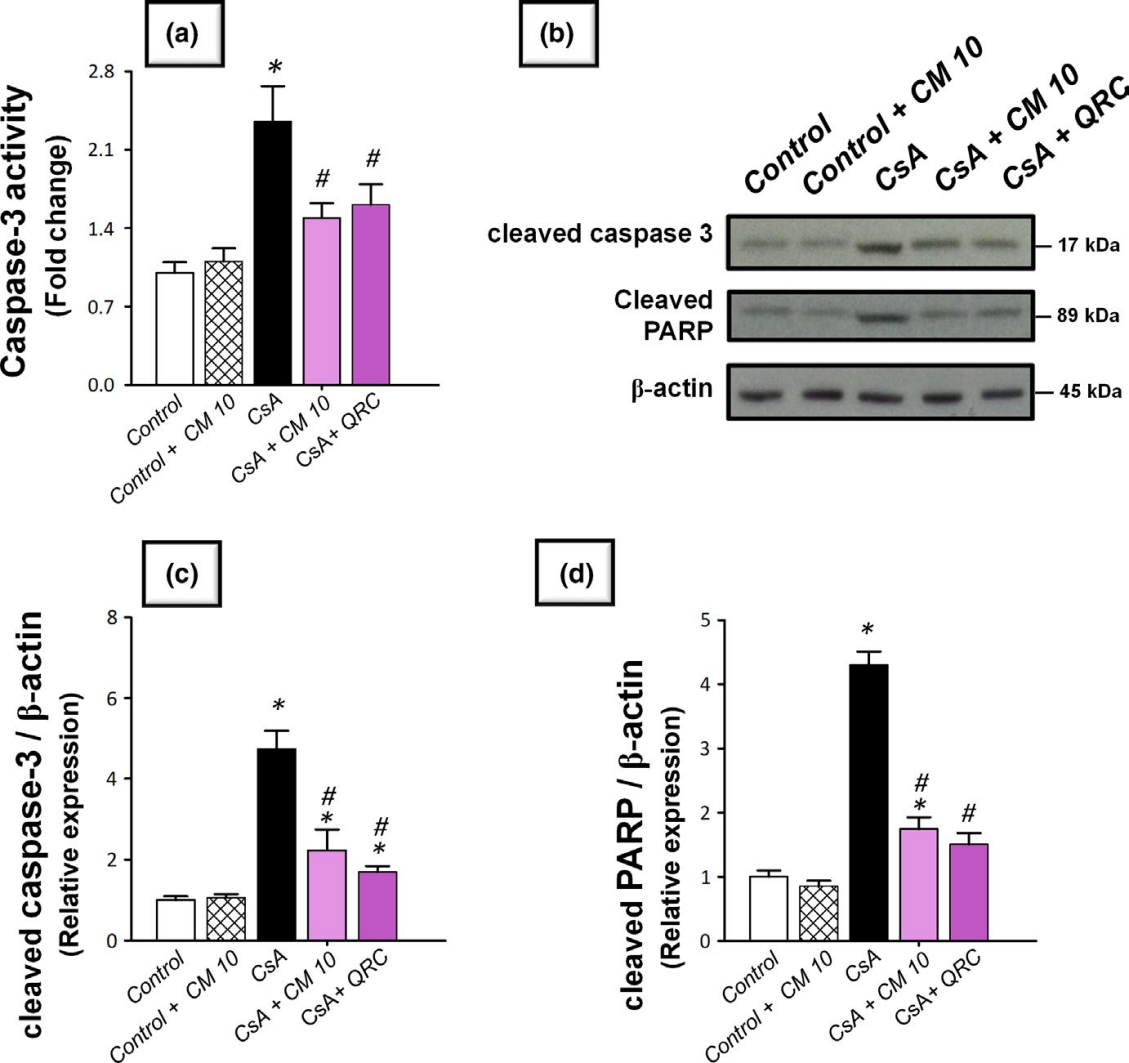
Camel milk counteracts the renal pro‐apoptotic events in cyclosporine‐induced nephrotoxicity in rats. (a) The activity of caspase‐3 enzyme. (b) Representative Western blots that depict the lowering effect of camel milk on the cleavage of caspase 3 and poly (ADP‐ribose) polymerase (PARP). (c) The analysis of cleaved caspase 3 relative expression. (d) The analysis of cleaved PARP relative expression. The values are mean ± *SEM* for three independent experiments per each group. *Significant versus control gp at *p* <.05; *^#^*Significance versus CsA‐treated gp at *p* <.05. CsA, Cyclosporine; CM 10, 10 ml kg^−1^ day^−1^ dose of camel milk; QRC, 50 mg kg^−1^ day^−1^ dose of the reference antioxidant quercetin

**FIGURE 6:**
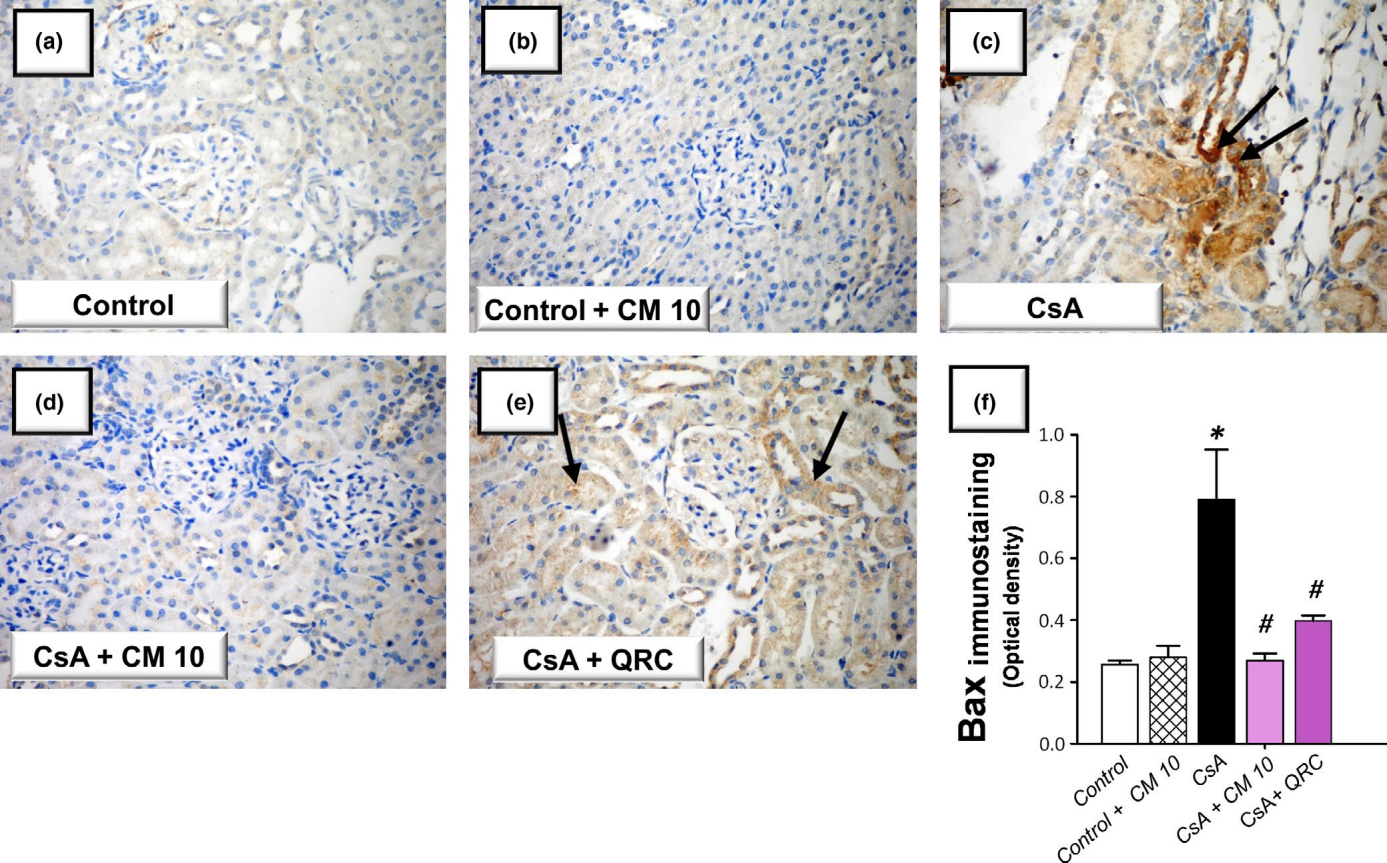
Camel milk lowers the renal expression of Bax in cyclosporine‐induced nephrotoxicity in rats. (a–e) Immunohistochemical labeling of Bcl‐2‐associated x protein (Bax). (f) Quantitative analysis of the optical density for Bax protein expression. The values are mean ± *SEM* for *n* = 4 per each group. *Significant versus control gp at *p* <.05; *^#^*Significance versus CsA‐treated gp at *p* <.05. CsA, Cyclosporine; CM 10, 10 ml kg^−1^ day^−1^ dose of camel milk; QRC, 50 mg kg^−1^ day^−1^ dose of the reference antioxidant quercetin

**FIGURE 7:**
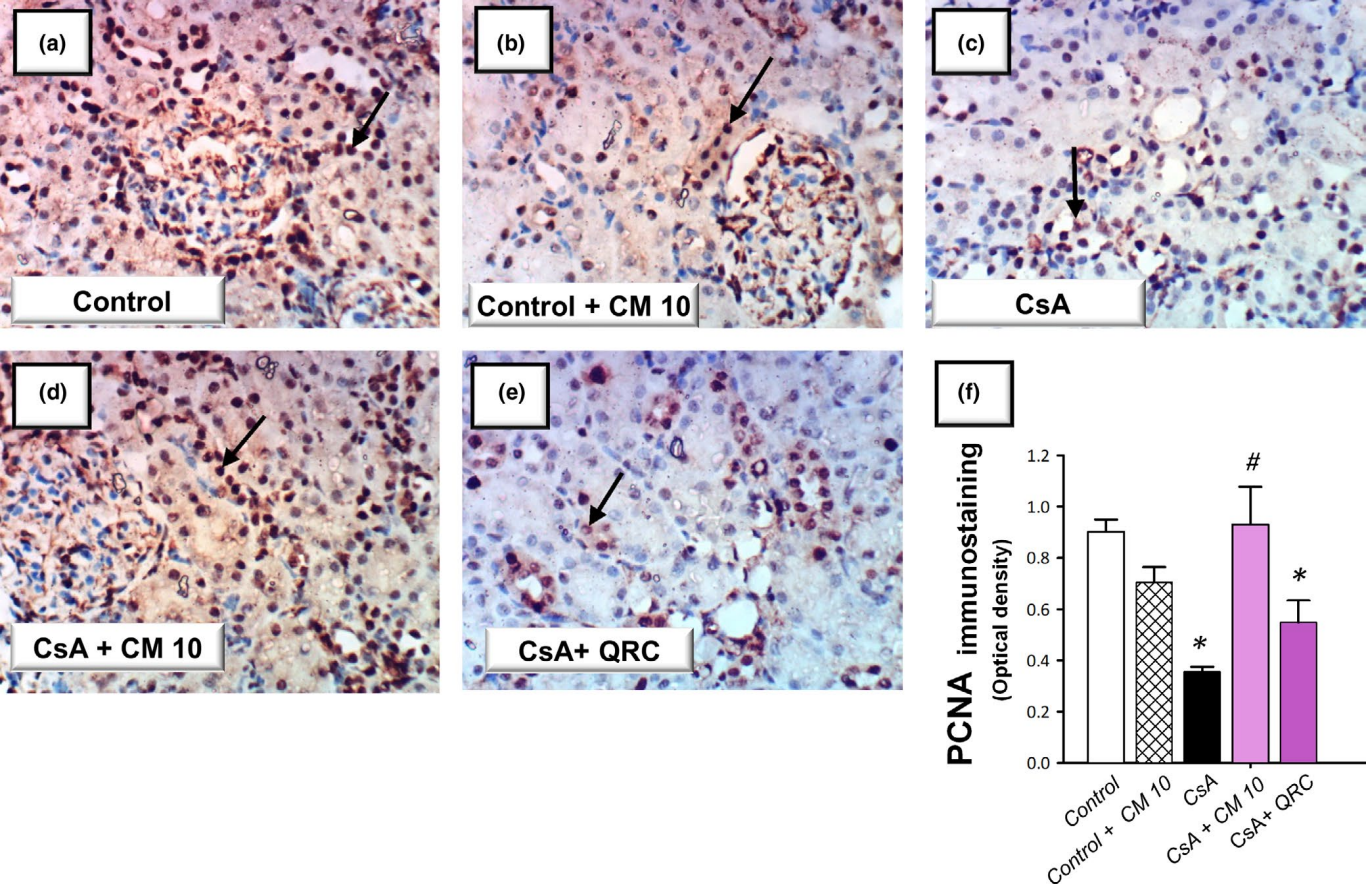
Camel milk enhances the expression of the proliferation signal PCNA in cyclosporine‐induced nephrotoxicity in rats. (a–e) Representative photomicrographs of the immunohistochemical labeling of the proliferating cell nuclear antigen (PCNA). (f) Quantitative analysis of the optical density for PCNA protein expression. The data are mean ± *SEM* for *n* = 4 per each group. *Significant versus control gp at *p* <.05; *^#^*Significance versus CsA‐treated gp at *p* <.05. CsA, Cyclosporine; CM 10, 10 ml kg^−1^ day^−1^ dose of camel milk; QRC, 50 mg kg^−1^ day^−1^ dose of the reference antioxidant quercetin

The apoptotic events triggered by CsA administration were investigated by detecting several apoptotic markers, including the cleavage of poly (ADP‐ribose) polymerase (PARP) and caspase‐3 by Western blotting together with the activity of caspase‐3. Meanwhile, the protein expression of the pro‐apoptotic Bax and the prosurvival PCNA proteins were analyzed by immunohistochemistry. As demonstrated in Figure 5, the renal tissues suffered a marked increase of the apoptotic events in response to CsA insult, as evidenced by significantly increased caspase‐3 activity (2.4‐fold) alongside an increased cleavage of caspase‐3 (4.7‐fold) and PARP (4.3‐fold) proteins. Meanwhile, CsA significantly elevated the protein expression of Bax pro‐apoptotic signal (3.08‐fold) and downregulated the protein expression of PCNA (0.39‐fold), relative to the control group (Figures 6 and 7). These aberrations were counteracted by camel milk administration that lowered the pro‐apoptotic signals and upregulated the protein expression of the proliferation signal PCNA, signifying enhanced renal cell survival.

### 3.5 Camel milk targets the activation of AKT/eNOS/NO pathway for combating apoptosis

**FIGURE 8:**
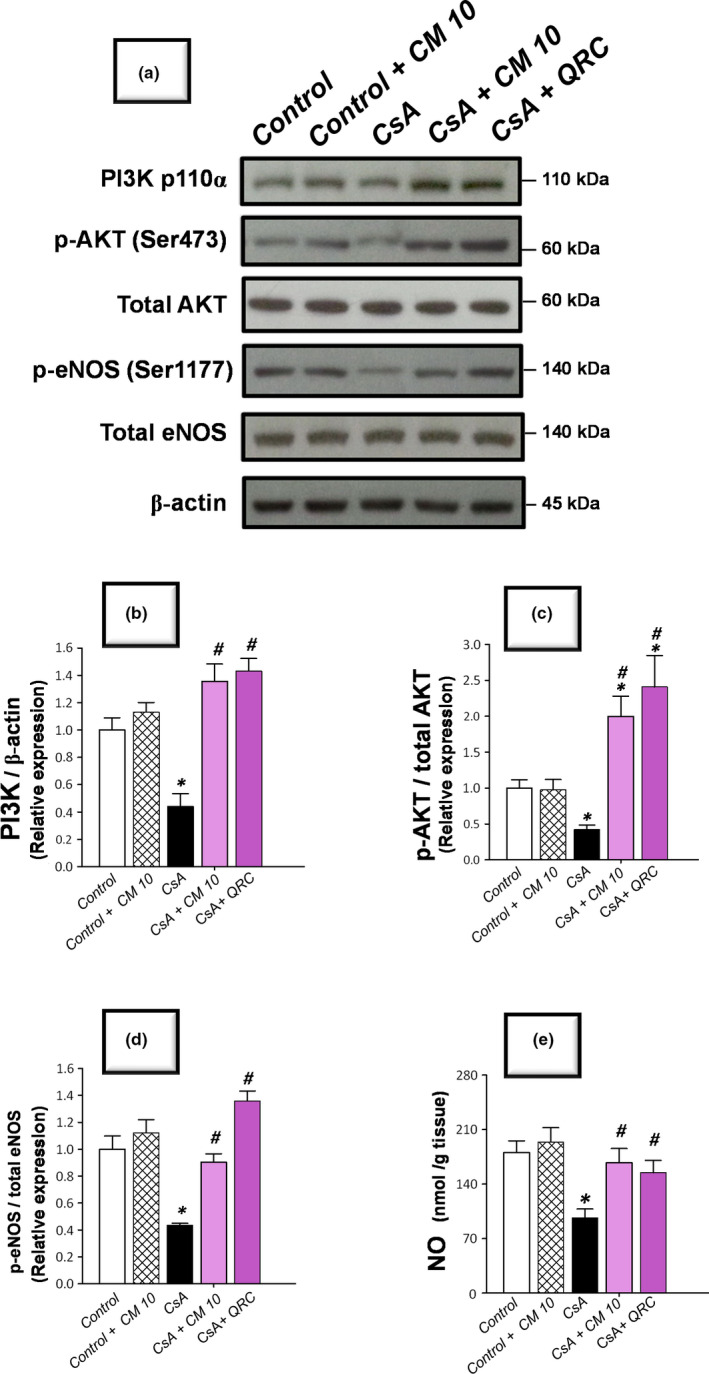
Camel milk upregulates the expression of PI3K, p‐AKT, and p‐eNOS and augments the nitric oxide levels in cyclosporine‐induced nephrotoxicity in rats. (a) Representative Western blots that depict the activation of PI3K/AKT/eNOS/NO pathway via upregulating the expression of PI3Kp110 (upper panel) and the phosphorylation of AKT (Ser473; middle panel), and p‐eNOS (Ser1177; lower panel). (b) PI3Kp110 relative protein expression. (c) p‐AKT(Ser473)/total AKT protein expression. (d) p‐eNOS (Ser1177)/total eNOS protein expression. The blotting values are mean ± *SEM* for three independent experiments per each group. (e) The levels of renal nitric oxide levels (NO). For NO levels, the values are mean ± *SEM* for *n* = 8 per each group. *Significant versus control gp at *p* <.05; *^#^*Significance versus CsA‐treated gp at *p* <.05. CsA, cyclosporine; CM 10, 10 ml kg^−1^ day^−1^ dose of camel milk; QRC, 50 mg kg^−1^ day^−1^ dose of the reference antioxidant quercetin

Evolving evidence has demonstrated that the activation of the AKT/eNOS/NO pathway exerts a dampening impact against renal apoptotic cell death in several toxicant‐induced renal pathologies (Arab et al., 2018a,b; Havasi & Borkan, 2011); however, the involvement of this pathway in the pathogenesis of CsA‐induced nephrotoxicity is inadequately defined. Hence, the signaling of the PI3K/AKT/eNOS/NO pathway was explored by detecting the immunoblotting of PI3K expression, along with the p‐AKT(Ser473)/total AKT, and p‐eNOS (Ser1177)/total eNOS ratios alongside renal levels of NO. As demonstrated in Figure 8, the renal tissues manifested a marked inhibition of the AKT/eNOS/NO pathway in response to CsA insult, as evidenced by a significant downregulation of PI3Kp110, p‐AKT(Ser473)/total AKT, and p‐eNOS (Ser1177)/total eNOS relative expression to reach 0.44‐fold, 0.42‐fold, and 0.43‐fold, respectively, relative to the control group. Meanwhile, the renal NO levels were significantly lowered to reach 0.54‐fold, relative to the control group. Consistent with the dampening of the apoptotic cell death, camel milk reversed the aforementioned changes, resulting in the activation of AKT/eNOS/NO pathway and mitigation of CsA‐evoked renal apoptosis and nephrotoxicity.

## 4 DISCUSSION

**FIGURE 9:**
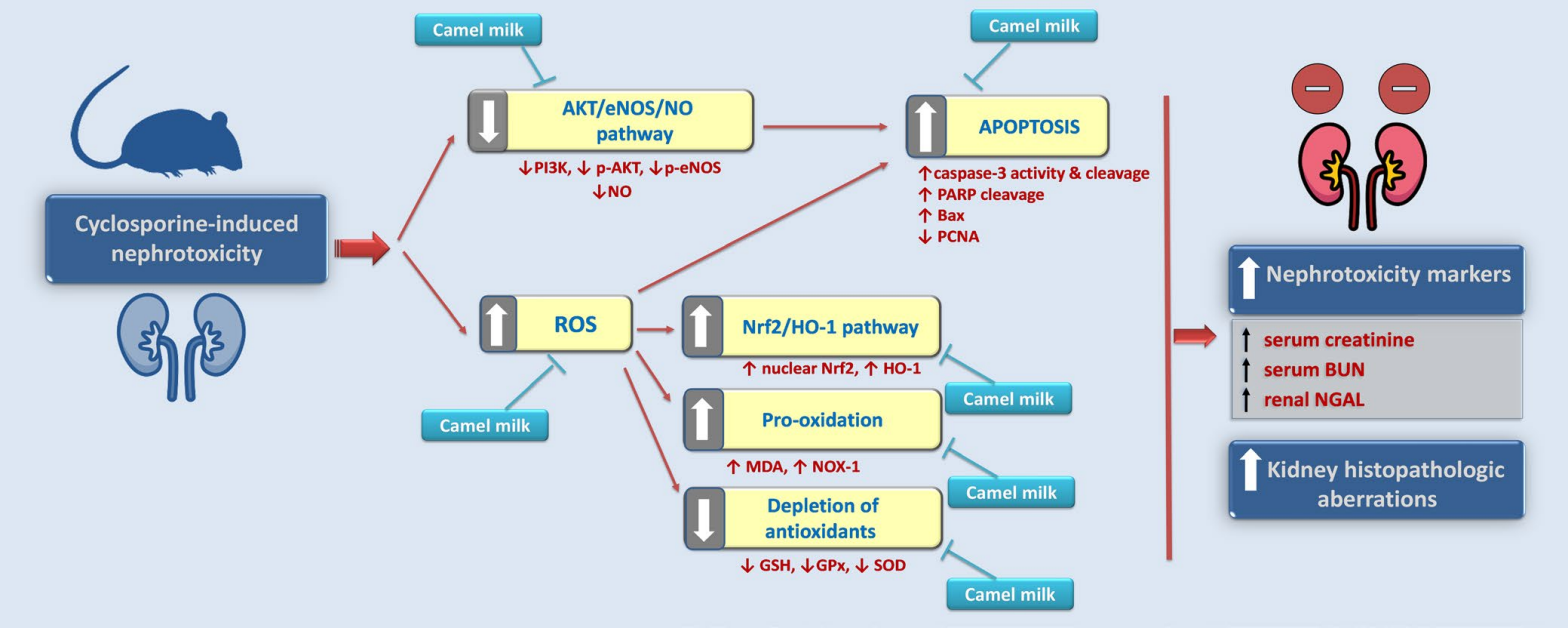
A concise summary of the implicated molecular mechanisms that mediated the ameliorative effects of camel milk in cyclosporine‐induced nephrotoxicity in rats. The present study reveals that camel milk attenuates cyclosporine‐induced nephrotoxicity through (a) Suppression of renal oxidative stress, as demonstrated by lowering MDA and the pro‐oxidant NOX‐1. The curtailing of the oxidative stress resulted in the restoration of Nrf2/HO‐1 pathway that was otherwise upregulated as a compensatory mechanism in response to the cyclosporine insult. (b) Augmentation of the antioxidant machinery, as evidenced by increasing the levels of GSH, GPx, and SOD. (c) Activation of the upstream AKT/eNOS/NO pathway, as evidenced by upregulated expression of PI3K, p‐AKT(Ser473)/total AKT, and p‐eNOS (Ser1177)/total eNOS ratios and increased levels of the vasodilator NO. (d) Suppression of the oxidative stress and activation of the AKT/eNOS/NO pathway, at least in part, converged for the dampening of renal cellular apoptosis, as proven with lowered caspase‐3 cleavage/activity and downregulated expression of the pro‐apoptotic Bax, with upregulation of the proliferation signal PCNA. The solid arrow (activation); blunt arrow (inhibition)

The present data reveal marked ameliorative effects of camel milk on CsA‐induced nephrotoxicity that were interceded by combating the oxidative aberrations, restoration of Nrf2/HO‐1 pathway, and boosting of the renal antioxidant machinery (Figure 9). In addition, camel milk counteracted the apoptotic events and activated the AKT/eNOS/NO pathway, culminating in lowering the renal injury evoked by CsA.

Ample evidence has demonstrated an increased incidence of CsA‐induced nephrotoxicity in the clinical setting in patients with solid‐organ transplantation and autoimmune disorders (Wu et al., 2018). Likewise, the rodent models have confirmed the marked nephrotoxicity triggered by CsA, which undermines the renal functions (Ateyya, 2015; El‐Sheikh et al., 2019). In the present work, CsA evoked a marked nephrotoxicity, as evidenced by increased serum levels of creatinine and BUN classical nephrotoxicity markers, alongside an increased renal NGAL protein expression. Notably, NGAL has been implicated in various renal aspects including cellular proliferation, differentiation, re‐epithelialization, and apoptosis besides its role as a sensitive, specific, and extremely predictive and early marker for renal tubular injury (Mori et al., 2005). Of note, camel milk administration elicited an obvious lowering of these renal nephrotoxicity markers, which is consistent with the reported renoprotective effects of camel milk in diabetes mellitus‐(Ayoub et al., 2018) and several toxicant‐induced renal injuries (Arab et al., 2018a,b; Ayoub et al., 2018).

Evolving lines of evidence have indicated that oxidative stress plays a crucial role in the pathogenesis of CsA‐induced nephrotoxicity (Abd‐Elhakim et al., 2020; Capasso et al., 2008; El‐Sheikh et al., 2019; Hasan et al., 2020). CsA has been reported to generate a surplus of ROS/free radicals due to its direct effect as uncoupler to the electron transport chain and during its metabolism by the cytochrome P450 3A enzyme (Origlia et al., 2006; Wu et al., 2018). In this context, CsA has been demonstrated to incur a marked in vivo oxidative stress in rodent models of nephrotoxicity by increasing the production of superoxide anions, hydrogen peroxide, peroxynitrite, and hydroxyl radicals. Likewise, the in vitro evidence has also reported that CsA increases the production of ROS in human renal mesangial cells (El‐Sheikh et al., 2019; Wu et al., 2018). The exaggerated overshooting of free radicals and ROS has been characterized to trigger lipid peroxidation of the renal cell membranes resulting in membrane derangements/dysfunction, as proved herein, and in previous reports (Ateyya, 2015; El‐Sheikh et al., 2019; Vangaveti et al., 2021). In the same regard, CsA has been reported to deplete the renal enzymatic/non‐enzymatic antioxidant armory, as seen herein, where CsA diminished the GSH, GPx, and SOD; observations that are in line with several reports (Ateyya, 2015; El‐Sheikh et al., 2019; Wu et al., 2018). In fact, the depletion of GSH has been reported to disrupt the activity of GPx and sensitize the renal cells to diverse stresses, which contributes to the aggravation of renal oxidative stress (Arab et al., 2018b). Of note, the observed upregulation of NOX‐1 has been reported to mediate the renal injury in several toxicant‐induced renal pathologies (Arab et al., 2018a,b; El‐Naga, 2014; Vangaveti et al., 2021). In the current set of experiments, CsA triggered an upregulated expression of Nrf2 and its downstream HO‐1 target. This response may be regarded as a compensatory mechanism of the renal cells to guard against the overshooting of CsA‐induced ROS production. These findings are in harmony with previous reports that CsA triggers the activation of Nrf2 signaling in tubular epithelial cells (Hamon et al., 2014; Wu et al., 2018).

Interestingly, camel milk administration attenuated the renal nephrotoxicity together with the oxidative stress markers, as evidenced by lowering NOX‐1 and lipid peroxides and augmenting the antioxidant defenses; cellular events that resulted in the restoration of Nrf2/HO‐1 pathway back to the normal state. These observations emphasize the marked antioxidant features of camel milk which were reported in diverse rodent models of renal pathologies (Arab et al., 2018a,b) and other experimental models (Abd‐Elhakim et al., 2020; Arab et al., 2014; Ayoub et al., 2018). Mechanistically, the rich content of antioxidants in camel milk has been reported to guard against ROS production. In this context, camel milk has a high content of ascorbic acid and tocopherol alongside antioxidant trace elements, such as selenium and zinc, essential components of GPx and SOD, respectively (Abd‐Elhakim et al., 2020; Ayoub et al., 2018). Additionally, the unique component lactoferrin plays a pivotal role in oxidative stress suppression by scavenging iron and curtailing the hydroxyl radical production (Abd‐Elhakim et al., 2020; Ayoub et al., 2018; Shori, 2015). Since free radicals and ROS production are the main triggers for Nrf2/HO‐1 pathway activation, the observed camel milk‐evoked suppression of renal oxidative stress markers and augmentation of renal antioxidant defenses may explain the observed restoration of the Nrf2/HO‐1 pathway back to the basal state (Wu et al., 2018).

Ample evidence exists that CsA triggers a marked renal tubular apoptotic cell death in murine models of CsA‐induced nephrotoxicity in vivo and in the in vitro models (De Arriba et al., 2013; Wu et al., 2018). In this context, De Arriba et al. (2013) have reported that CsA instigates a marked generation of ROS that lowers the mitochondrial membrane potential and triggers the leakage of cytochrome c through the mitochondrial permeability transition pores, thereby, initiating a cascade of pro‐apoptotic events. As a result, activation of caspase 9 is ensued which activates the executioner caspase 3‐induced DNA fragmentation (Havasi & Borkan, 2011). These events are in accordance with the current findings that CsA provoked an increased caspase 3 activity/cleavage, cleavage of PARP, upregulated expression of the pro‐apoptotic Bax signal and downregulated the proliferation signal PCNA. Equally important, CsA inactivated the anti‐apoptotic AKT/eNOS/NO pathway and lowered the renal content of NO. Classically, the AKT/eNOS/NO is an anti‐apoptotic pathway that advocates renal cell survival (Hasan et al., 2020; Havasi & Borkan, 2011). The observed inactivation of AKT/eNOS/NO pathway is compliant with the former literature that reported PI3K/AKT downregulation in response to superfluous ROS production (Guo et al., 2015; Hasan et al., 2020). Mechanistically, activated p‐AKT (Ser473) has been reported to instigate the phosphorylation and inactivation of Bax and Bad pro‐apoptotic signals (Hasan et al., 2020; Havasi & Borkan, 2011; Liu et al., 2020).

Notably, camel milk suppressed the renal apoptotic events, activated the AKT/eNOS/NO pathway, and augmented the renal NO levels. This was evidenced by lowering of caspase‐3, PARP, and Bax pro‐apoptotic signals, along with increasing the expression of PI3Kp110, p‐AKT(Ser473)/total AKT, and p‐eNOS (Ser1177)/total eNOS. These findings agree with the reported anti‐apoptotic features of camel milk in diabetic lymphoid organs (Sayed et al., 2017), colitis, and toxicant‐induced renal pathologies (Arab et al., 2018a,b). Conceptually, modalities that can suppress the renal apoptotic cell death have been regarded as promising agents for favoring renal recovery and preserving kidney function (De Arriba et al., 2013; El‐Sheikh et al., 2019; Hasan et al., 2020; Havasi & Borkan, 2011). In this context, diverse antioxidant agents—such as erdosteine, schisandrin, apocynin, and spirulina—have been characterized as effective interventions against CsA‐evoked nephrotoxicity in rodents, a notion that coincides with the present study findings (Wu et al., 2018). The plausible mechanism by which camel milk counteracts CsA‐triggered pro‐apoptotic events is the antioxidant/radical scavenging features of camel milk which lower ROS levels; the main triggers that provoke apoptotic cell death (Abd‐Elhakim et al., 2020; Havasi & Borkan, 2011; Mohamed et al., 2019). Additionally, the attenuation of the renal apoptosis can be, at least in part, indirectly driven by the activation of AKT/eNOS/NO pathway (Havasi & Borkan, 2011; C. Liu et al., 2020). This notion is corroborated by the finding that camel milk whey protein increases the phosphorylation/activation of AKT in wounds of diabetic mice (Badr, 2012). A unique observation in the present study is the ability of camel milk to boost the renal NO levels, which contributes to the attenuation of CsA‐induced nephrotoxicity. In fact, increased NO content can counteract the renal vasoconstriction, which is a hallmark for CsA‐induced renal injury (El‐Bassossy & Eid, 2018; Wu et al., 2018). Additionally, NO has been reported to combat the renal interstitial inflammatory cell invasion, extracellular matrix protein deposition, and glomerular thrombosis/ischemia (Wu et al., 2018).

## 5 CONCLUSION

The present findings have demonstrated that camel milk may be a promising candidate for the alleviation of CsA‐induced nephrotoxicity. These favorable effects were mediated, at least in part, through the suppression of renal oxidative stress, enhancement of antioxidants, and curtailing of apoptosis by targeting Nrf2/HO‐1 and AKT/eNOS/NO pathways. Further studies are warranted to delineate the exact underlying molecular mechanisms and the implicated signaling.

## CONFLICT OF INTEREST

No conflict of interest is declared by the authors.
